# Organic or Inorganic Nitrogen and Rhizobia Inoculation Provide Synergistic Growth Response of a Leguminous Forb and Tree

**DOI:** 10.3389/fpls.2019.01308

**Published:** 2019-10-22

**Authors:** Peng Zhang, R. Kasten Dumroese, Jeremiah R. Pinto

**Affiliations:** ^1^Key Laboratory of Sustainable Forest Ecosystem Management-Ministry of Education, School of Forestry, Northeast Forestry University, Harbin, China; ^2^Rocky Mountain Research Station, Forest Service, U.S. Department of Agriculture, Moscow, ID, United States

**Keywords:** amino acid, arginine, inorganic nitrogen, isotopic nitrogen, *Lupinus latifolius*, nitrogen fixation, organic nitrogen, *Robinia pseudoacacia*

## Abstract

Our objective was to better understand how organic and inorganic nitrogen (N) forms supplied to a tree, *Robinia pseudoacacia*, and a perennial forb, *Lupinus latifolius*, affected plant growth and performance of their symbiotic, N-fixing rhizobia. In one experiment, we tested five sources of N [none; three inorganic forms (ammonium, nitrate, ammonium-nitrate); and an organic form (arginine)] in combination with or without rhizobia inoculation. We measured seedling morphology, allometry, nodule biomass, and N status. A second experiment explored combinations of supplied ^15^N and inoculation to examine if inorganic or organic N was deleterious to nodule N-fixation. Plant growth was similar among N forms. A positive response of nodule biomass to N was greater in *Robinia* than *Lupinus*. For *Robinia*, inorganic ammonium promoted more nodule biomass than organic arginine. N-fixation was concurrent with robust supply of either inorganic or organic N, and N supply and inoculation significantly interacted to enhance growth of *Robinia*. For *Lupinus*, the main effects of inoculation and N supply increased growth but no interaction was observed. Our results indicate that these important restoration species for forest ecosystems respond well to organic or inorganic N forms (or various forms of inorganic N), suggest that the nodulation response may depend on plant species, and show that, in terms of plant growth, N supply and nodulation can be synergistic.

## Introduction

Nitrogen (N) has long been demonstrated as the most critical element for enhanced productivity of plant growth. Soil N is present in inorganic forms, such as ammonium and nitrate, and organic forms, such as amino acids (Christou et al., 2006). Ammonium and nitrate are the predominant inorganic N forms usually taken up by plants (Hawkins and Robbins, 2010). Studies confirmed, however, that amino acids sometimes serve as a primary N source for vegetation (Kielland, 1994; Persson and Näsholm, 2001; Inselsbacher and Näsholm, 2012; Whiteside et al., 2012; Wei et al., 2015) and plants have a reduced carbon cost when assimilating organic N into proteins (Franklin et al., 2017). Organic N can be an efficient and environmentally favorable N source (Öhlund and Näsholm, 2002).

Plants may show different growth responses to the form of N they are provided. For trees, Bigg and Daniel (1978) and Yao et al. (2011) collectively examined six conifer species and found that three species grow better with ammonium than nitrate (*Picea engelmannii*, *Pinus contorta*, *Pinus yunnanensis*), two grow better with nitrate than ammonium (*Pseudotsuga menziesii*, *Pinus densata*), and one had no preference (*Pinus tabuliformis*). *Picea abies* and *Pinus sylvestris* seedlings, with an ability to uptake intact amino acids, grow as well as, or better, when they are supplied with amino acids compared to seedlings with access to inorganic N sources (Öhlund and Näsholm, 2001; Gruffman et al., 2012). Conversely, a field study with *Abies fraseri*, *Pinus resinosa*, and *Populus nigra* × *Populus maximowiczii* found that seedling growth and foliar N response resulting after an amino acid supply was similar to inorganic N applications only when the organic form was applied at rates two or three times higher than that of the inorganic form (Wilson et al., 2013). The N form provided in the growth substrate affects not only plant growth but also plant biomass allocation. Amino acids were suggested to have a direct positive effect on root growth of the small herb *Arabidopsis thaliana* cultivated in a sterile agar growth system (Cambui et al., 2011). *Picea abies* and *P. sylvestris* seedlings given organic N (arginine) in a container nursery had larger root systems and a greater root-to-shoot ratio than did seedlings with access to inorganic N (Gruffman et al., 2012).

Inoculation of seeds with rhizobia is known to increase nodulation, leading to enhanced N content, growth, and yield of legume crops (Adgo and Schulze, 2002; Gan et al., 2010; Namvar et al., 2011) and woody species (Dan et al., 1993; Diabate et al., 2005; Jayakumar and Tan, 2006; Dumroese et al., 2009). The effectiveness of inoculation on nodulation and subsequent plant growth response can be affected by N fertilization, but the conclusions are mixed. While broadly concluded that mineral soil N [nitrate (NO_3_^−^) and ammonium (NH_4_^+^)] inhibits nodulation, nodule growth, and activity in legumes (Imsande, 1986; Streeter and Wong, 1988; Guo et al., 1992; Bollman and Vessey, 2006; Camargos and Sodek, 2010), studies have shown that low (Rohm and Werner, 1991; Vessey and Waterer, 1992; Gulden and Vessey, 1997; Goicoechea et al., 2005; Yashima et al., 2005) and moderate (Peoples et al., 1995; Bado et al., 2006) supplies of N can stimulate nodulation and N fixation. Nodulation with apparent N-fixing ability has also been noted when N was supplied in high amounts (Dumroese et al., 2009). These differences may be a function of either the symbiosis or the N source.

The response of legumes to N level is species specific and depends upon the particular rhizobia-legume symbiosis (Manhart and Wong, 1980; Harper and Gibson, 1983). Nodules of *Lupinus angustifolius* infected with *Rhizobium* sp. 127E15 were unaffected by addition of 15 mM NO_3_^−^, whereas all other combinations of rhizobia–*L. angustifolius* and rhizobia–*Vigna unguiculata* averaged a 30% reduction in nodule biomass (Manhart and Wong, 1980). Nitrate was found to induce an inhibitory effect on nodulation and N fixation in pea plants at levels as low as 0.1 mM, while the level at which NH_4_^+^ became inhibitory was 1.0 mM and higher (Rys and Phung, 1984; Silsbury et al., 1986; Waterer and Vessey, 1993a, Waterer and Vessey, 1993b; Svenning et al., 1996). While sensitivity to NO_3_^−^ has been found to occur in all legume species studied so far (Streeter and Wong, 1988; Lucinski et al., 2002; Fei and Vessey, 2003), the impact of NH_4_^+^ on symbiosis is weaker than that observed for NO_3_^−^ (Bollman and Vessey, 2006). Streeter and Wong (1988) noted that nitrate may affect the symbiotic process by reducing nodule formation, reducing nitrogenase activity, or by affecting the ratio of nodule dry mass to whole plant mass. These lines of research continue (Lucinski et al., 2002) but with increasing attention to molecular changes that influence N-fixation brought about by NO_3_^−^ (e.g., Valkov et al., 2017). Despite this intensive effort, the combined effects of N and rhizobia inoculation on nonagronomic legumes have received little attention. To our knowledge, no studies have examined the effects of organic N on nodule formation and subsequent plant growth.

In this study, we assessed the plant response of two Fabaceae (legume) species supplied with different sources of N and rhizobia inoculation in a controlled greenhouse system. *Lupinus latifolius*, hereafter lupine, is a native perennial forb occurring in forests of western North America from British Columbia to Baja California to New Mexico. It has a bushy, densely branched growth habit originating from a woody caudex (Antos and Halpern, 1997). It is commonly used for rehabilitation of disturbed forest sites and for erosion control because it grows well on droughty and/or low-fertility sites, has a deep root system for stabilizing soil, and forms associations with N-fixing microorganisms (Halverson et al., 1986; Doede, 2005). *Robinia pseudoacacia*, hereafter black locust, is a medium-sized tree native to the southeastern United States that has been widely planted and naturalized elsewhere in temperate North America, Europe, southern Africa, and Asia (Olson and Karrfalt, 2008). In the United States, it has been used for afforestation of abandoned farmlands, timber, erosion control, and soil stabilization on disturbed sites (Keresztesi, 1980; Roberts et al., 1983). Both species are commonly grown as container seedlings in some nurseries in North America, and so determining proper N supply and inoculation regimes will undoubtedly have a significant impact on the availability of these two species for restoration activities.

Therefore, our goal was to investigate the growth and allometry, physiological responses, and nodulation status of lupine and black locust provided N in organic and inorganic forms, with or without rhizobia inoculation. To the best of our knowledge, this is the first study to examine the effects of supplying organic N to seedlings and subsequent rhizobia nodule development and function. Arginine was chosen as our model amino acid partly because it had been used in other studies (Öhlund and Näsholm, 2001; Öhlund and Näsholm, 2002; Persson et al., 2003; Öhlund and Näsholm, 2004; Gruffman et al., 2013; Wilson et al., 2013) and partly because of its chemical properties. Arginine is an N-rich molecule containing four N atoms, which collectively contribute 32% of its molecular weight. It is positively charged and thus acts as a basic cation in soils with acidic pH, binding to negatively charged soil particles, which restricts its mobility. Our objectives were to see whether (1) seedlings supplied with organic and inorganic N forms have similar growth; (2) inoculated and noninoculated seedlings supplied with organic and inorganic N forms have similar rhizobia nodule biomass; and (3) high rates of organic and inorganic N supply discourage N fixation.

## Methods and Materials

### Species Selection and Seed Treatment

To meet our objectives, we grew lupine (collected in 2010 at 919 m elevation on the Newport Ranger District, Colville National Forest, Washington, USA) and black locust (collected in 2012 in Kentucky, USA; Lawyer Nursery, Inc., Plains, Montana, USA). Seeds were treated with hot water scarification before sowing. Lupine seeds were submerged 1 min in 100°C water (seeds:water = 10 g:800 ml), immediately soaked in running tap water for 48 h, and stratified 15 days at 3°C. Black locust seeds were submerged in 70°C water (seeds:water = 15 g:500 ml), the heat source was immediately removed, and the seeds and water were allowed to cool for 24 h. Expanded seeds of each species were sown.

### Experiment One—Seedling and Nodule Growth

#### Design and Seed Sowing

Our first experiment was a completely randomized design with two independent factors: rhizobia inoculation and N source. We employed two levels of rhizobia inoculation (control or inoculated) and five sources of N: none; three inorganic forms [ammonium (NH_4_^+^), nitrate (NO_3_^−^), ammonium-nitrate (NH_4_NO_3_)]; and an organic form (L-arginine; Sigma-Aldrich Co., St. Louis, Missouri, USA). At the time of planting, one half of the seeds was inoculated with either lupine- or black locust-appropriate rhizobia. The lupine inoculant included three proprietary isolates, whereas black locust was a combination of a proprietary isolate and USDA 3436 (Plant Probiotics Company, Indianapolis, Indiana, USA). For each inocula replication, we slightly moistened the scarified seeds, applied each peat-based inocula at a rate of 1 g (∼3 × 10^8^ CFUs) per 100 g of seeds, thoroughly mixed the seeds and inocula, and immediately hand-planted 3 seeds into Ray Leach^TM^ Super Cells (164 cm^3^, 3.8-cm diameter, 21-cm depth; Stuewe & Sons, Inc., Tangent, Oregon, USA) filled with a 1:1 (v:v) mix of Sphagnum peatmoss:vermiculite (Forestry #1, SunGro Horticulture Canada Ltd., Canada). Sown seeds were covered with 1 cm of coarse grit to reduce evaporation and cryptogam growth. We planted eight cells for each species, inoculation, and N source combination (each seedling served as a replicate). Sown cells were randomly placed onto a table inside a fully controlled greenhouse at the U.S. Department of Agriculture, Forest Service, Rocky Mountain Research Station in Moscow, Idaho (46.7232°, −117.0029°; 798 m elevation), thinned to a single seedling after germination, and rearranged during each irrigation to minimize edge effects.

#### Seedling Culture

Seedling irrigation needs were determined gravimetrically for each species, inoculation, and N source combination. When mass of the water reached 75% of the total water mass at container capacity, seedlings were irrigated with just enough water to return the cells to container capacity (Dumroese et al., 2015); this occurred one or more times each week. Fourteen days after planting (DAP), we began supplying N. Each lupine seedling received a total of 6 mg N during a 6-week period (1 mg per week; 14 to 56 DAP), whereas black locust seedlings each received a total of 24 mg N during a 12-week period (2 mg per week; 14 to 98 DAP). We used three different nutrient stock solutions, differing only with respect to the chemical form of N (**Table 1**). Each stock solution also included micronutrients [sulfur (S), 13%; manganese (Mn), 8%; iron (Fe), 7.5%; copper (Cu), 2.3%; boron (B), 1.35%; molybdenum (Mo), 0.04%; Peters Professional® S.T.E.M.^TM^, the Scotts Company, Marysville, Ohio, USA] at 15 mg L^−1^ and Sprint 330 [10% Fe (chelated); Becker Underwood, Inc., Ames, Iowa, USA] at 20 mg L^−1^. Once each week, when gravimetric weights indicated irrigation was needed, we calculated the amount of water required to replenish the medium for each seedling to container capacity. This value was always >15 ml seedling^−1^. Starting with 15 ml of stock solution, we added the appropriate N source at either 1 (lupine) or 2 (black locust) mg N and applied the subsequent solution to an individual seedling *via* syringe. As required, we then added plain water to reach the total irrigation amount calculated for each seedling based on the gravimetric measurements. The resulting solutions had 67 (lupine) and 134 (black locust) mg N L^−1^ with concentrations of phosphorus (P), potassium (K), S, calcium (Ca), and magnesium (Mg) provided at ratios of 1.0, 0.7, 1.1, 0.9, and 1.2 to 1.0 N (**Table 1**). We reinoculated seedlings 28 DAP to ensure inoculation; 0.5 g of inoculant was diluted in 1.25 L water and then 5 ml of inoculant solution (2 mg inoculant) was applied to each seedling.

**Table 1:** Nutrient stock solutions for application of different nitrogen sources.

Stock nutrient solution components			Nitrogen sources				
			(NH_4_)_2_SO_4_	Ca(NO_3_)_2_	NH_4_NO_3_	Arginine	None
	Solution number =		1	2	3	3	3
		N^z^	P	K	S	Ca	Mg
			——————————————–—————— Chemicals (10^−3^ mol L^−1^) ————————————————————				
Lupine							
	(NH_4_)_2_SO_4_		2.42	–	–	–	–
	Ca(NO_3_)_2_		–	2.40	–	–	–
	NH_4_NO_3_		–	–	2.46	–	–
	C_6_H_14_N_4_O_2_		–	–	–	1.19	–
	75% H_3_PO_4_		1.95	1.95	1.95	1.95	1.95
	KH_2_PO_4_		0.34	0.34	0.34	0.34	0.34
	KCl		0.90	0.90	0.90	0.90	0.90
	CaCl_2_		2.41	–	2.41	2.41	2.41
	MgSO_4_		–	2.42	2.42	2.42	2.42
	MgCl_2_		2.46	–	–	–	–
					
Black locust							
	(NH_4_)_2_SO_4_		4.80	–	–	–	–
	Ca(NO_3_)_2_		–	4.77	–	–	–
	NH_4_NO_3_		–	–	4.89	–	–
	C_6_H_14_N_4_O_2_		–	–	–	2.38	–
	75% H_3_PO_4_		1.95	1.95	1.95	1.95	1.95
	KH_2_PO_4_		2.33	2.33	2.33	2.33	2.33
	CaCl_2_		4.76	–	4.76	4.76	4.76
	MgSO_4_		–	4.75	4.75	4.75	4.75
	MgCl_2_		4.88	–	–	–	–
		——————————————————————— Applied element concentration (mg L^−1^) ————————————————————————					
Lupine		67	70	46	79	96	59
Black locust		133	140	91	154	190	114

^z^Control seedlings received no nitrogen but the other nutrients at these rates.

#### Seedling Measurements

For lupine, the longest petiole length and the widest leaf width were measured weekly from 28 DAP until harvest 56 DAP. For black locust, seedling height and root-collar diameter (RCD) were measured every 14 days from 42 DAP until harvest 112 DAP. At harvest, we gently washed root systems of both species free of medium and counted and harvested nodules. Shoots (leaf and stems for block locust), roots, and nodules were separated and oven-dried 72 h at 65°C for biomass determination. The oven dried shoots and roots for each treatment were subsequently ground to pass a 0.44-mm mesh and analyzed for N concentration by dry combustion in a LECO CN600 (LECO Corp., St. Joseph, Michigan, USA).

### Experiment Two—Nodule Activity

Based on our results from the first experiment, we sowed a second crop of each species as described above (except for N source, total N applied, and growing time) to measure differential dilution of absorbed ^15^N-labeled fertilizer by N inputs through N-fixation by rhizobia (Hardarson and Danso, 1993). Because preliminary data analyses revealed no significant differences among inorganic N forms for seedling growth, we supplied inorganic N only as NH_4_NO_3_ amended with NH_4_NO_3_ enriched to 98 atom % ^15^N (Sigma Aldrich, St. Louis, Missouri, USA). Organic N was supplied as arginine hydrochloride amended with L-arginine-α-hydrochloride enriched to 98 atom % ^15^N (Cambridge Isotope Laboratory, Inc., Tewksbury, Massachusetts, USA). The ^15^N:^14^N was about 0.0037:1. Irrigation and N were supplied as described above, but this time, the black locust seedlings were only treated for 8 weeks (16 mg N total).

Individual seedlings were harvested 56 DAP (lupine) and 70 DAP (black locust). Seedlings were washed free of growing media and rinsed in deionized water, partitioned into leaves, stems, and roots, then oven dried 48 h at 60°C, weighed, and ground as previously described to determine biomass. At least five seedlings from each species × N combination were analyzed for isotopic N. The concentrations of ^15^N and ^14^N in each tissue were determined with a continuous flow isotope ratio mass spectrometer (Finnigan Delta PlusXP, Thermo Fisher Scientific, Breman, Germany) at the Washington State University Stable Isotope Core Laboratory (Pullman, Washington, USA). Nitrogen isotope ratio was expressed as follows:

$$\delta^{15}\text{N}=\left(\left[{}^{15}\text{N sample}{/}^{14}\text{N sample}\right]/\left[{}^{15}\text{N air}{/}^{14}\text{N air}\right]-1\right)\times 1,000$$

### Statistical Analysis

Both experiments were analyzed as factorial designs. Analysis of variance (two-way ANOVA) was used to examine the effects of the independent variables (N, rhizobia inoculation) and their interaction on final seedling petiole length (cm); leaf width (mm); height (cm); RCD (mm); dry biomass (g) of leaves, stems, shoots, roots, and nodules; ratio of shoot dry biomass to root dry biomass (S:R); N concentration (g kg^−1^) and content (mg kg^−1^) of leaves, stems, shoots, and roots; and δ^15^N. Residual plots were used to assure data met model assumptions for homoscedasticity and normality. We used Tukey’s HSD for *post hoc* tests of differences between model means. Results were considered significant at α = 0.05. Box-plot visualizations were created using SigmaPlot (version 13.0; Systat Software, San Jose, California, USA).

## Results

### Lupine

**Figure 1:**
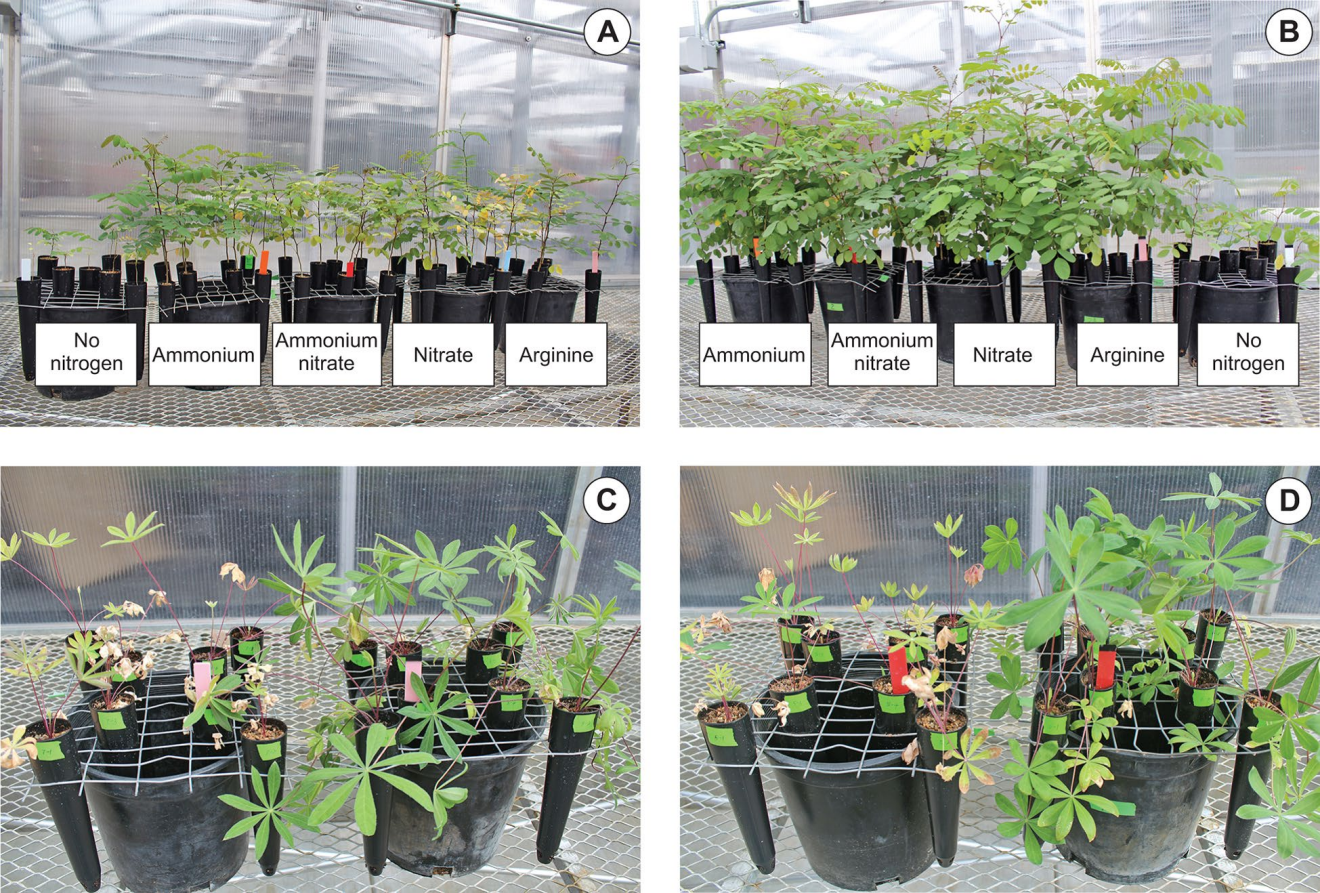
Growth responses of black locust and lupine seedlings (98 and 56 days after planting, respectively) supplied inorganic or organic nitrogen with or without rhizobia inoculation and grown in a greenhouse. Noninoculated **(A)** and inoculated **(B)** black locust seedlings. Lupine seedlings fertilized with arginine (left plants noninoculated, right plants inoculated) **(C)** or ammonium-nitrate (left plants noninoculated, right plants inoculated) **(D)**.

In the first experiment, none of the final measured response variables were affected by an N × inoculation interaction. While N overall was a significant factor, only the presence of N (as opposed to no N) significantly affected every measured response variable except shoot-to-root ratio (S:R) and nodule biomass (**Table 2**). Seedlings supplied with N had significantly longer petioles and greater leaf width, and a trend toward greater shoot and root biomass, than those not supplied N. Inoculation significantly affected every final measured response variable (**Table 2**; **Figure 1**). Inoculated seedlings had significantly longer petioles and wider leaves, as well as significantly more shoot, root, and nodule biomass than the noninoculated seedlings, but noninoculated seedlings had a significantly higher S:R.

**Table 2:** Mean morphological parameters of lupine seedlings inoculated (I) with (+) or without (−) rhizobia and supplied with different nitrogen (N) sources [none, ammonium (NH_4_), nitrate (NO_3_), ammonium nitrate (NH_4_NO_3_), or arginine (A)] for 6 weeks (n = 8). Main effect (ME) means are presented in italics. Different letters within a dependent variable indicate significant differences (α = 0.05) using Tukey’s HSD.

Dependent variable	I	ME	Nitrogen sources					ME	Sources of variation		
									*P* values		
			None	NH_4_	NO_3_	NH_4_NO_3_	A	*I*	N	I	N × I
Petiole length (cm)	+		12.5	15.9	15.1	15.6	15.6	*14.9 a*		**<0.0001**	
	−		8.1	10.6	12.2	12.1	11.4	*10.9 b*			0.8686
		*N*	*10.3 b*	*13.3 ab*	*13.6 a*	*13.9 a*	*13.5 a*		**<0.0096**		
Leaf width (mm)	+		8.2	9.6	9.6	10.8	10.7	*9.8 a*		**<0.0001**	
	−		4.2	6.2	6.2	6.3	5.4	*5.7 b*			0.3253
		*N*	*6.2 b*	*7.9 a*	*7.9 a*	*8.5 a*	*8.1 a*		**0.0005**		
Shoot biomass (g)	+		0.39	0.51	0.52	0.74	0.67	*0.57 a*		**<0.0001**	
	−		0.06	0.17	0.20	0.19	0.16	*0.16 b*			0.0544
		*N*	*0.23 b*	*0.34 ab*	*0.36 ab*	*0.47 a*	*0.42 a*		**0.0002**		
Root biomass (g)	+		0.17	0.23	0.22	0.30	0.27	*0.24 a*		**<0.0001**	
	−		0.02	0.05	0.07	0.06	0.05	*0.05 b*			0.2813
		*N*	*0.09 b*	*0.14 ab*	*0.15 ab*	*0.18 a*	*0.16 ab*		**0.0082**		
Nodule biomass (g)	+		0.049	0.055	0.063	0.080	0.070	*0.063 a*		**<0.0001**	
	−		0.002	0.002	0.009	0.005	0.000	*0.004 b*			0.1575
		*N*	*0.025*	*0.028*	*0.036*	*0.042*	*0.035*		0.0796		
Shoot:root	+		2.9	2.4	2.3	2.6	2.6	*2.5 a*		**0.0001**	
	−		4.5	3.9	3.0	4.0	4.0	*3.9 b*			0.9342
		*N*	*3.7*	*3.2*	*2.6*	*3.3*	*3.3*		0.3985		

Nitrogen significantly affected shoot and root N concentration and content (**Table 3**). The shoot N concentration was largest for seedlings supplied with inorganic N forms, with arginine and the no-N treatment having the lowest concentrations. Inorganic NH_4_^+^ and NH_4_NO_3_ had significantly greater root N concentration than seedlings not supplied N, whereas NO_3_^−^ and arginine were intermediate, yielding results similar to the no-N treatment and the other inorganic sources. Seedlings supplied inorganic and organic N had significantly more shoot N content than no-N seedlings. The largest root N content was provided by NH_4_NO_3_ and was significantly more than the no-N treatment, while NH_4_^+^, NO_3_^−^, and arginine were intermediate, yielding results similar to the no-N treatment and NH_4_NO_3_. Inoculation significantly affected shoot and root N concentration and N content (**Table 3**). Inoculated seedlings had 2×, 1.5×, 10×, and 7× the shoot N concentration, root N concentration, shoot N content, and root N content of the noninoculated seedlings.

**Table 3:** Mean nitrogen (N) concentrations and contents of lupine seedlings inoculated (I) with (+) or without (−) rhizobia and supplied with different N sources [none, ammonium (NH_4_), nitrate (NO_3_), ammonium nitrate (NH_4_NO_3_), or arginine (A)] for 6 weeks (n = 8). Main effect (ME) means are presented in italics. Different letters within a dependent variable indicate significant differences (α = 0.05) using Tukey’s HSD.

Dependent variable		I	ME	Nitrogen sources					ME	Sources of variation		
										*P* values		
				None	NH_4_	NO_3_	NH_4_NO_3_	A	*I*	N	I	N × I
N concentration (mg g^−1^)												
	Shoot	+		27.5	38.9	38.8	33.3	28.2	33.3 a		** <0.0001**	
		−		11.7	15.8	14.8	16.9	11.9	*14.2 b*			0.0642
			*N*	*19.6 b*	*27.3 a*	*26.8 a*	*25.1 a*	*20.0 b*		**0.0001**		
	Root	+		24.1	32.3	31.0	31.3	27.2	*29.2 a*		** <0.0001**	
		−		15.7	21.4	20.3	21.9	19.9	*19.8 b*			0.7584
			*N*	*22.4 b*	*26.8 a*	*25.7 ab*	*26.6 a*	*23.5 ab*		**0.0095**		
N content (mg seedling^−1^)												
	Shoot	+		11.1	20.1	19.9	24.7	19.0	*18.9 a*		** <0.0001**	
		−		0.7	2.7	3.1	3.3	1.9	*2.3 b*			0.1121
			*N*	*5.9 b*	*11.4 a*	*11.5 a*	*14.0 a*	*10.4 a*		**0.0050**		
	Root	+		4.2	7.6	6.9	9.4	7.4	*7.1 a*		** <0.0001**	
		−		0.2	1.0	1.5	1.4	0.9	*1.0 b*			0.1620
			*N*	*3.4 b*	*4.3 ab*	*4.2 ab*	*5.4 a*	*4.2 ab*		**0.0146**		

**Figure 2:**
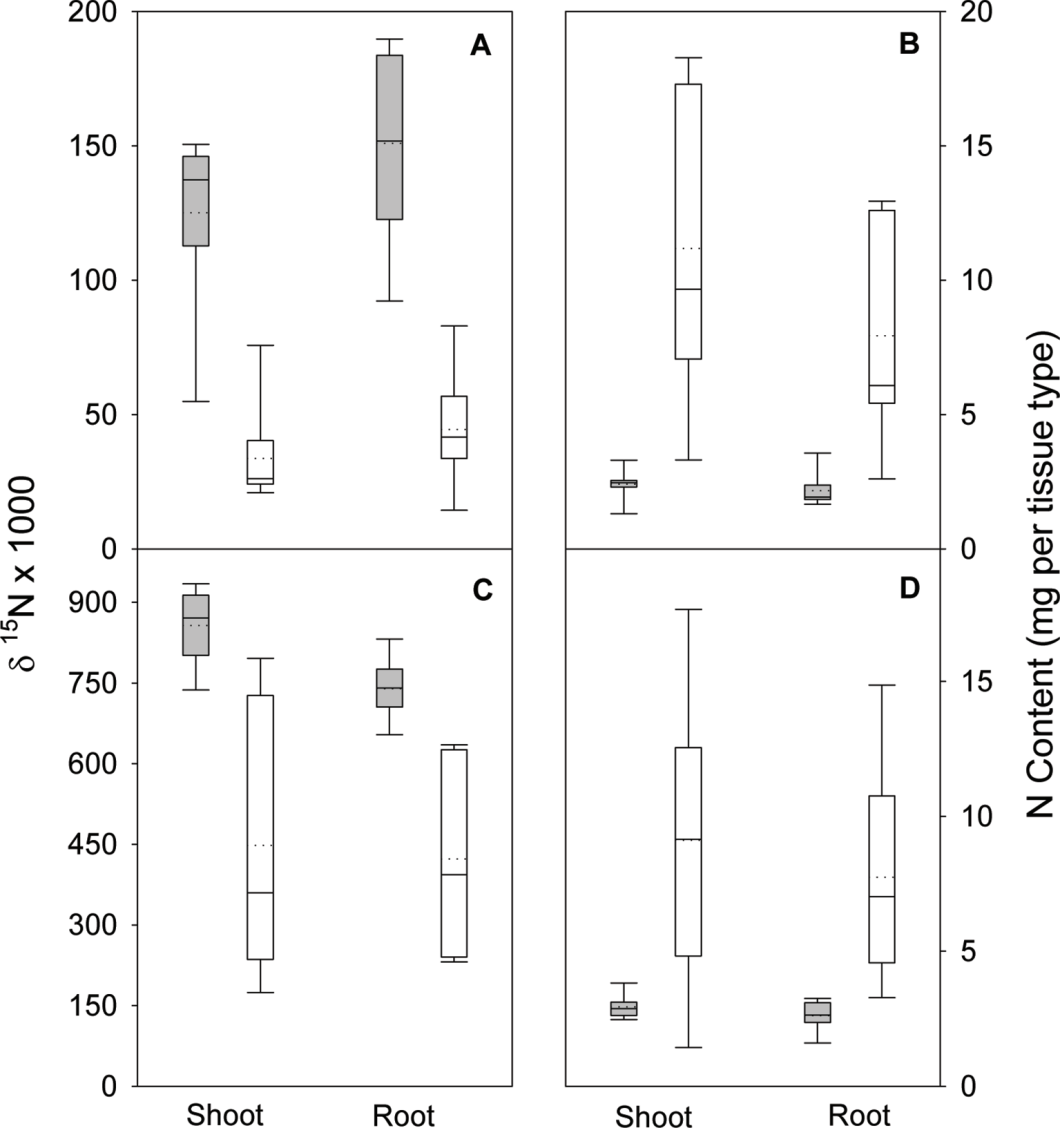
Nitrogen isotope ratio (δ^15^N) and N content in shoots and roots of lupine seedlings 56 days after planting in the greenhouse. Seedlings were supplied with either arginine **(A** and **B)** or ammonium-nitrate **(C** and **D)** after either being inoculated (white boxes) or not inoculated (gray boxes) with rhizobia. Vertical boxes represent approximately 50% of the observations and lines extending from each box are the upper and lower 25% of the distribution. The solid horizontal line in the center of each box is the median value and the dotted line is the mean.

In the nodule activity experiment, no interaction of N form (arginine and NH_4_NO_3_) and inoculation was observed for shoot, root, or total seedling biomass. Although N form was not significant for any biomass parameter, inoculation was (all p ≤ 0.0088). For arginine and NH_4_NO_3_, including inoculation increased total biomass (**Figure 1**). N form and inoculation significantly interacted (all p ≤ 0.0051) to affect shoot, root, and total seedling N isotope ratio (δ^15^N); δ^15^N values were lower when seedlings were inoculated but the magnitude of difference was greater in seedlings supplied arginine than for those supplied NH_4_NO_3_ (**Figure 2**). No significant N form × inoculation interaction was observed for shoot or root N content and the main effect of N form was not significant. Inoculation was, however, significant for N content of shoot and root (p < 0.0001), with inoculated seedlings having significantly more N (**Figure 2**).

### Black Locust

In the first experiment, N and inoculation significantly interacted to affect final measurements of seedling height, leaf biomass, stem biomass, shoot biomass, and root biomass (**Table 4**). The pattern of the interaction was orderly and similar: without inoculation, seedlings supplied N were taller and had greater biomass than those not supplied N; adding inoculation resulted in additional growth to seedlings either supplied N or not supplied N, but the increase in growth was much greater for those supplied N (**Table 4**; **Figure 1**).

**Table 4:** Mean morphological parameters of black locust seedlings inoculated (I) with (+) or without (−) rhizobia and supplied with different nitrogen (N) sources [none, ammonium (NH_4_), nitrate (NO_3_), ammonium nitrate (NH_4_NO_3_), or arginine (A)] for 12 weeks (n = 8). Main effect (ME) means are presented in italics. Different letters within a dependent variable indicate significant differences (α = 0.05) using Tukey’s HSD.

Dependent variable	I	ME	Nitrogen sources					ME	Sources of variation		
									*P* values		
			None	NH_4_	NO_3_	NH_4_NO_3_	A	*I*	N	I	N × I
Height (cm)	+		11.4 d	47.6 a	45.0 ab	39.4 b	40.8 ab	*36.8*		** <0.0001**	
	−		6.6 d	22.7 c	24.1 c	20.3 c	20.7 c	*18.9*			**0.0006**
		*N*	*9.0*	*35.2*	*34.5*	*29.8*	*30.8*		** <0.0001**		
Root-collar diameter (mm)	+		3.04	6.83	6.64	6.64	6.61	*5.95 a*		** <0.0001**	
	−		1.54	4.51	4.39	4.19	3.90	*3.70 b*			0.3226
		*N*	*2.29 b*	*5.67 a*	*5.52 a*	*5.41 a*	*5.25 a*		** <0.0001**		
Leaf biomass (g)	+		0.73 bc	4.42 a	4.60 a	4.16 a	4.20 a	*3.62*		** <0.0001**	
	−		0.12 c	1.70 b	1.41 b	1.17 bc	1.26 b	*1.13*			**0.0029**
		*N*	*0.43*	*3.06*	*3.01*	*2.67*	*2.73*		** <0.0001**		
Stem biomass (g)	+		0.28 cd	3.49 a	3.14 ab	2.78 b	2.69 b	*2.47*		** <0.0001**	
	−		0.05 d	0.84 c	0.97 c	0.79 c	0.72 c	*0.67*			** <0.0001**
		*N*	*0.16*	*2.17*	*2.05*	*1.78*	*1.70*		** <0.0001**		
Shoot biomass (g)	+		1.01 bc	7.91 a	7.74 a	6.94 a	6.89 a	*6.10*		** <0.0001**	
	−		0.17 c	2.54 b	2.38 b	1.96 b	1.97 b	*1.80*			**0.0003**
		*N*	*0.59*	*5.22*	*5.06*	*4.45*	*4.43*		** <0.0001**		
Root biomass (g)	+		0.75 cd	4.94 a	3.91 b	4.10 ab	3.66 b	*3.47*		** <0.0001**	
	−		0.09 d	1.47 c	1.76 c	1.40 c	1.25 c	*1.19*			**0.0027**
		*N*	*0.42*	*3.20*	*2.84*	*2.75*	*2.45*		** <0.0001**		
Nodule biomass (g)	+		0.09 ef	0.46 a	0.36 ab	0.26 bcd	0.30 bc	*0.30*		** <0.0001**	
	−		0.02 f	0.20 cde	0.14 def	0.17 cde	0.11 ef	*0.13*			0.1588
		*N*	*0.05*	*0.33*	*0.25*	*0.22*	*0.20*		** <0.0001**		
Shoot:root	+		1.9	1.6	2.0	1.7	2.1	*1.9*		0.1846	
	−		2.3	1.7	1.5	1.5	1.6	*1.7*			0.1499
		*N*	*2.1*	*1.6*	*1.8*	*1.6*	*1.9*		0.1165		

Nitrogen significantly affected every final measured response variable except S:R (**Table 4**). In general, N-supplied seedlings had significantly more height, RCD, leaf biomass, and stem, shoot, nodule, and root biomass than seedlings not supplied N, but values among N forms were similar. For N-supplied seedlings, N form did not affect seedling size but it did significantly affect nodule biomass, with NH_4_^+^ producing the most, followed by NO_3_^−^, NH_4_NO_3_, and arginine; the no-N treatment yielded the least amount of nodule biomass. Similar to N, inoculation significantly affected every final measured response variable except S:R (**Table 4**), with inoculated seedlings having greater values than their noninoculated cohorts.

The N × inoculation interaction was significant for leaf N concentration (**Table 5**). Noninoculated and inoculated seedlings grown without N had similar leaf N concentration, but for seedlings supplied with N, noninoculated seedlings had significantly greater N concentration than inoculated ones. N significantly affected N concentration in leaves and stems but not roots: leaf N concentration was greatest for seedlings not supplied N followed by those supplied with NH_4_^+^ or NH_4_NO_3_, with those supplied with arginine or NO_3_^−^ producing the lowest leaf N concentration. In general, no-N seedlings had significantly larger stem N concentration than N-supplied seedlings, but seedling stem N concentrations for seedlings supplied with N were similar regardless of N form. Inoculation had no effect on N concentration of leaves, stems, or roots (**Table 5**).

**Table 5:** Mean nitrogen (N) concentrations and contents of black locust seedlings inoculated (I) with (+) or without (−) rhizobia and supplied with different nitrogen (N) sources [none, ammonium (NH_4_), nitrate (NO_3_), ammonium nitrate (NH_4_NO_3_), or arginine (A)] for 12 weeks (n = 8). Main effect (ME) means are presented in italics. Different letters within a dependent variable indicate significant differences (ခα = 0.05) using Tukey’s HSD.

Dependent variable		I	ME	Nitrogen sources					ME	Sources of variation		
										*P* values		
				None	NH_4_	NO_3_	NH_4_NO_3_	A	*I*	N	I	N × I
N concentration (mg g^−1^)												
											
	Leaf	+		32.4 a	27.9 a	28.6 a	29.5 a	28.2 a	*29.8*		0.3936	
		−		34.4 a	32.0 a	20.9 b	30.9 a	28.2 a	*28.6*			**0.0365**
			*N*	*33.1*	*30.0*	*24.7*	*30.2*	*28.2*		**0.0098**		
	Stem	+		28.0	20.1	22.0	23.2	24.3	*23.6*		0.1709	
		−		26.9	21.9	16.4	24.8	20.3	*21.7*			0.2169
			*N*	*26.7 a*	*21.0 b*	*19.2 b*	*24.0 b*	*22.3 b*		**0.0362**		
	Root	+		27.4	26.8	31.6	31.2	30.3	*29.3*		0.1444	
		−		27.6	29.6	24.0	26.7	23.7	*26.2*			0.5865
			*N*	*27.3*	*28.9*	*27.0*	*28.2*	*27.3*		0.9633		
N content (mg seedling^−1^)												
	Leaf	+		24.4	123.6	131.3	122.6	118.3	*104.1 a*		** <0.0001**	
		−		4.3	55.4	32.6	36.7	38.2	*33.4 b*			0.0526
			*N*	*14.4 b*	*89.5 a*	*82.0 a*	*79.7 a*	*78.2 a*		** <0.0001**		
	Stem	+		9.3	70.4	69.4	64.9	64.1	*55.4 a*		** <0.0001**	
		−		2.6	19.8	17.3	20.0	15.9	*14.8 b*			0.0564
			*N*	*4.6 b*	*45.1 a*	*43.4 a*	*42.5 a*	*40.0 a*		**0.0002**		
	Root	+		21.8 ab	101.9 a	89.7 ab	101.9 a	88.3 ab	*80.7*		** <0.0001**	
		−		7.6 b	76.3 ab	64.4 ab	57.3 ab	37.4 ab	*28.6*			**0.0061**
			*N*	*10.7*	*94.3*	*75.5*	*76.2*	*68.4*		** <0.0001**		

Nitrogen and inoculation interacted to affect root N content (**Table 5**). The pattern followed the interaction observed for seedling growth: without N, noninoculated and inoculated seedlings had the same root N content, whereas with N, inoculated seedlings had more N than their noninoculated cohorts. Nitrogen significantly affected leaf, stem, and root N content. In general, N-supplied seedlings had significantly higher N content in leaves, stems, and roots than those not receiving N, but seedling N content was similar regardless of N form. Inoculation significantly increased N content of leaves, stems, and roots (**Table 5**).

**Figure 3:**
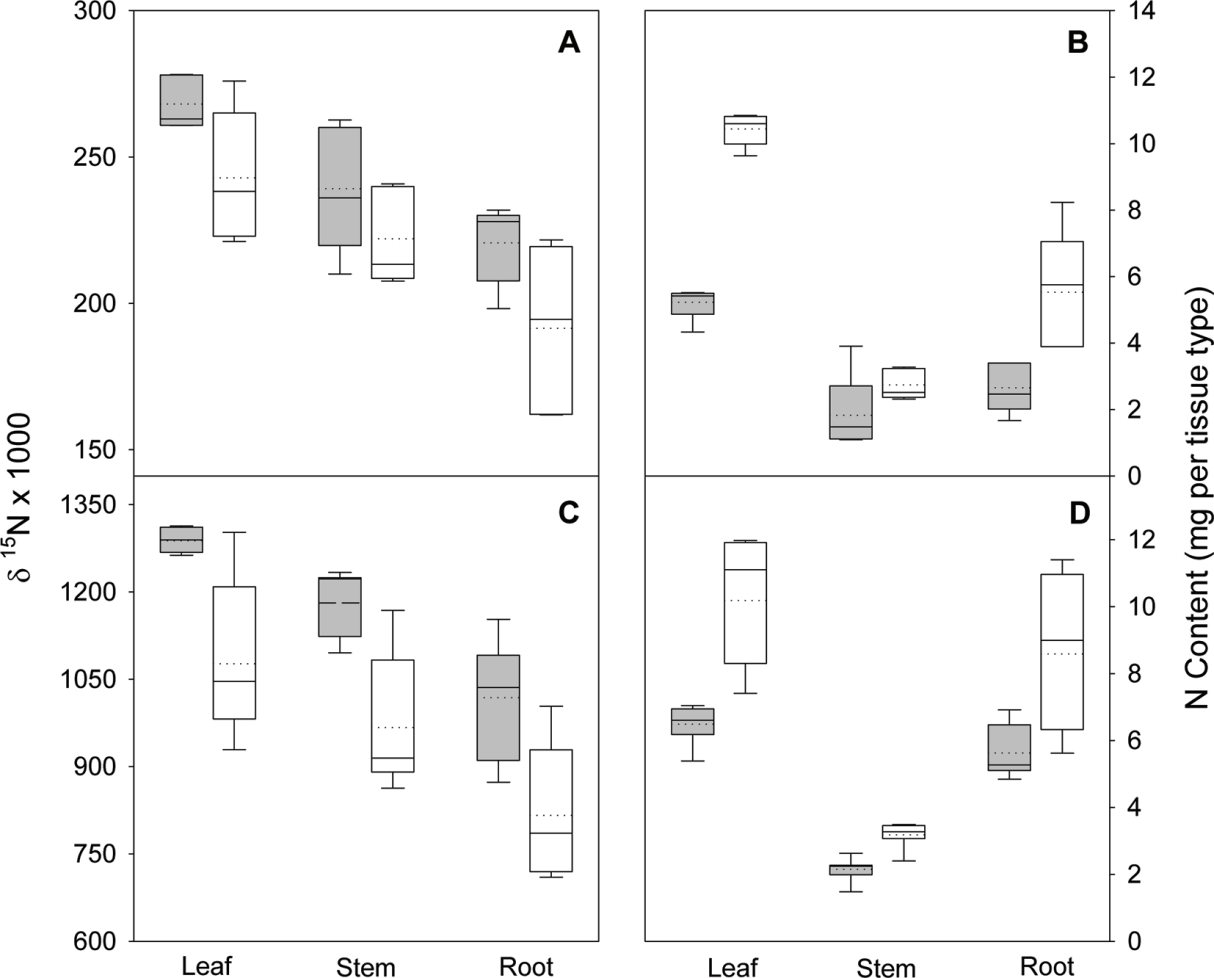
Nitrogen isotope ratio (δ^15^N) and N content in leaves, stems, and roots of black locust seedlings 70 days after planting in the greenhouse. Seedlings were supplied with either arginine **(A** and **B)** or ammonium-nitrate **(C** and **D)** after either being inoculated (white boxes) or not inoculated (gray boxes) with rhizobia. Vertical boxes represent approximately 50% of the observations and lines extending from each box are the upper and lower 25% of the distribution. The solid horizontal line in the center of each box is the median value and the dotted line is the mean.

In the nodule activity experiment, no interaction of N form (arginine and NH_4_NO_3_) and inoculation was observed for shoot, root, or total seedling biomass, but N form and inoculation were both significant (p ≤ 0.0151), except for stem biomass. Regardless of N form, inoculation increased total biomass about 35%. The interaction of N form and inoculation was significant for all tissue types and total seedling δ^15^N (all p ≤ 0.0212); δ^15^N decreased with inoculation (**Figure 3**). For N content, the interaction was not significant for any tissue type or total seedling, N form had no effect on total seedling N content, and inoculation significantly (p < 0.0013) increased N content (**Figure 3**).

## Discussion

In this study, noninoculated lupine and black locust seedlings supplied organic or inorganic N had similar growth; moreover, these seedlings grew significantly larger than their cohorts not supplied N (**Tables 2** and **4**). Inoculation with rhizobia, with or without N supply, improved seedling growth and increased N content compared to noninoculated seedlings (**Tables 2**‒**5**), similar to the response noted for other legume plants (Dan et al., 1993; Adgo and Schulze, 2002; Diabate et al., 2005; Jayakumar and Tan, 2006; Dumroese et al., 2009; Gan et al., 2010; Namvar et al., 2011). Inoculated black locust seedlings supplied N had 157% more biomass than those not supplied N (Uselman et al., 2000). We noted, however, that inoculation decreased the S:R for lupine seedlings (**Table 2**) but not for black locust seedlings (**Table 4**), suggesting that the subsequent allocation response of the plant to improved N status may be either species or plant form dependent. Although we observed minor and sporadic nodulation on noninoculated plants, we did not evaluate their N-fixing availability; other research suggests that these types of nodules may not actively fix atmospheric N (Tirichine et al., 2006).

It has long been proposed that mineral N inhibits nodulation, nodule growth, and activity in legumes (Imsande, 1986; Streeter and Wong, 1988; Guo et al., 1992; Bollman and Vessey, 2006; Camargos and Sodek, 2010). While low N rates improved nodule formation in black locust (Rohm and Werner, 1991), N rates exceeding 0.84 mg N week^−1^ reduced nodule number and nodule biomass (Roberts et al., 1983; Rohm and Werner, 1991). Our results, however, show that, although N form did not affect seedling size, N supplied at 2 mg N week^−1^ significantly enhanced nodule formation on black locust compared to seedlings not supplied N (**Table 4**); NH_4_^+^ seemed to favor nodulation the most, confirming that the impact of NH_4_^+^ on symbiosis is weaker than that observed for NO_3_^−^ (Bollman and Vessey, 2006). Our data suggests that more robust N supply may not preclude nodule formation, similar to results observed in some species (Peoples et al., 1995; Bado et al., 2006; Dumroese et al., 2009). A similar result was, however, not apparent on lupine seedlings (**Table 2**). Nodulation on lupine was unaffected by N form, but for black locust, N supplied as NH_4_^+^ was superior to arginine, suggesting that the nodulation response may be plant species dependent (Manhart and Wong, 1980; Harper and Gibson, 1983).

Although increasing N rates have been associated with decreasing nodule number on black locust (Roberts et al., 1983; Rohm and Werner, 1991), the nitrogenase activity of the nodules was either unaffected (Roberts et al., 1983) or increased (Rohm and Werner, 1991). Our results of the N isotope ratio in different parts of lupine and black locust seedlings supplied with organic or inorganic N showed that δ^15^N was decreased in different tissue types in inoculated seedlings relative to noninoculated seedlings, indicating dilution within the seedling caused by additions of atmospheric N-fixation within the nodules (Hardarson and Danso, 1993). Indeed, our seedlings had 3× (black locust; **Table 5**) to 9× (lupine; **Table 3**) more N content when inoculation was combined with additions of N.

Thus, it is not surprising that N supply and inoculation significantly interacted to affect morphology and root N content of black locust (**Tables 4** and **5**). The combination of inoculation and access to N promoted more growth (i.e., height, biomass, nodules) than did inoculation alone. Despite higher N contents in inoculated black locust seedlings supplied N, their leaf N concentration was lower than noninoculated seedlings supplied N. This lower N concentration is due to dilution (Salifu and Jacobs, 2006) that likely occurred in these rapidly growing plants during the three weeks between the final application and harvest. Thus, for black locust, the combination of inoculation and access to N improved growth more than either factor alone. Uselman et al. (2000) also found that the combination of inoculation and N supply produced larger seedlings than inoculation alone. For lupine, we were unable to detect a significant N supply by inoculation interaction (**Tables 2** and **3**) but N content data (**Table 3**) followed a pattern similar to that for black locust. For noninoculated black locust seedlings, we noted N contents that exceeded those expected by supply of N alone. The additional N may originate from the substrate we used. Peat initially contains appreciable N that may have subsequently been mineralized and available for plant uptake. In their review, Cheng and Kuzyakov, (2005) report that rapid soil wetting-drying cycles tend to increase mineral soil organic matter mineralization; this condition is common in small-volume seedling containers such as the ones we used, especially as the seedlings became larger (Landis et al., 1989). Moreover, the presence of roots can lead to a threefold increase in mineralization (Cheng and Kuzyakov, 2005).

Many plant species (e.g., Persson and Näsholm, 2001; Metcalfe et al., 2011), including trees (e.g., Wei et al., 2015; Uscola et al., 2017) show the capacity for intact uptake of amino acids, especially through their mycorrhizal networks (McFarland et al., 2010; Whiteside et al., 2012). Microbes utilize more amino acid N in the short term (e.g., Warren, 2009; Farrell et al., 2014). In the longer term (more than 2 days), seedlings may capitalize on the rapid cycling of amino acid N through the microbial biomass (Harrison et al., 2008; Warren, 2009), which may explain the results of studies in greenhouses showing that plants grown on amino acid N performed as well as those given inorganic N (Öhlund and Näsholm, 2001; Gruffman et al., 2012). Amino acid application in field settings, however, could alter the plant-microorganism balance due to an enhanced carbon environmnet (Schobert et al., 1988), leading to unexpected varations in N taken up by plants, which may explain the reduced growth with amino acids observed by Wilson et al. (2013). Our results showed that seedlings of both broadleaved plant species supplied with arginine grew as well as seedlings given inorganic N, although we did not measure if the organic N was taken up intact by either species.

Although amino acids are suggested to have a direct positive effect on root growth (Cambui et al., 2011), in our study, neither root growth or S:R of lupine or black locust seedlings were affected (**Tables 2** and **4**). The stimulatory effect on root growth is thought to be associated with development of mycorrhizal symbioses (Cambui et al., 2011), an aspect that we did not examine in our study.

Successful use of seedlings in forest restoration activities requires that they meet specific target criteria needed to overcome environmental limitations on their intended outplanting site (Dumroese et al., 2016). Fertilization in the nursery can provide a more rapid method for obtaining target plant size than the sole use of an N-fixing symbiont on a leguminous tree and can be done within current operational standards (Dumroese et al., 2009). This is important because nursery efficiency (e.g., plant growth per unit time) is one of many factors supporting successful nursery operation (Dumroese et al., 2011). In addition, Villar-Salvador et al. (2008) report that seedlings supplied high rates of fertilizer during nursery production grew larger and had improved morphological and physiological traits (i.e., greater biomass, root growth capacity, photosynthetic rates, and N concentrations) than their cohorts supplied low rates of fertilizer regardless of rhizobial inoculation; this translated into more survival and growth after outplanting. Our results demonstrate that robust fertilization need not sacrifice development of nodules capable of N-fixation.

In summary, we grew a forb and tree species using inorganic and organic N forms with and without genus-specific (lupine) or species-specific (black locust) rhizobia inoculation. We found that seedlings grew equally well with either N form; nodule biomass in lupine was unaffected by N form, whereas in black locust, nodule biomass in NH_4_^+^-supplied seedlings was greater than that in arginine-supplied seedlings, an apparent species-specific response; and that N-fixation readily occurred in the presence of high levels of N supply, leading to enhance seedling N content. Our results suggest that these potentially important species for forest restoration activities can be produced in nurseries to acceptable morphological targets using robust levels of either organic or inorganic forms of N and have functioning symbionts as well.

## Author Contributions

PZ, KD, and JP shared equally in conceiving the research, designing the experiment, interpreting results, and drafting the manuscript. RD and JP provided research funding and technical expertise in seedling production. PZ tended and sampled seedlings. KZ and JP performed the data analyses.

## Funding

This study was funded by the Fundamental Research Funds for the Central Universities (China; 2572019CP16), U.S. Department of Agriculture, Forest Service (USFS) Rocky Mountain Research Station (RMRS), and the USFS National Center for Reforestation, Nurseries, and Genetic Resources.

## Conflict of Interest

The authors declare that the research was conducted in the absence of any commercial or financial relationships that could be construed as a potential conflict of interest.
